# Enhancing multiple scales of seafloor biodiversity with mussel restoration

**DOI:** 10.1038/s41598-022-09132-w

**Published:** 2022-03-23

**Authors:** Mallory A. Sea, Jenny R. Hillman, Simon F. Thrush

**Affiliations:** Institute of Marine Science, University of Auckland, Auckland, 1142 New Zealand

**Keywords:** Biodiversity, Restoration ecology

## Abstract

Restoration projects are underway internationally in response to global declines in shellfish beds. As diverse biological assemblages underpin a variety of ecosystem services, understanding broader changes in biodiversity associated with mussel restoration becomes increasingly valuable to scientists and restoration practitioners. Studies generally show bivalve beds increase species richness and abundance, but results are scale-dependent and conditional on the mobility of specific communities observed. We examined biodiversity at multiple scales to determine how communities with varying levels of mobility are influenced by subtidal mussel restoration. Significant changes in assemblage structure were observed in both mobile fish and epifaunal communities, with enhanced species richness and total abundance of associated individuals. In contrast, we observed site-dependent effects of bivalve restoration on macrofaunal community structure and composition, with sheltered, harbour mussel bed communities numerically dominated by detritivores accustomed to organically enriched, muddy sediments. Sediment organic matter significantly increased within mussel beds, and distance-based linear models showed that sediment organic matter was an important predictor of macrofaunal assemblage structure on mussel beds, highlighting the significance of benthic-pelagic coupling and biodeposition to soft-sediment communities. This study contributes novel methods and ecological insights on the role of species mobility and site selection in structuring restoration outcomes, better informing future mussel restoration efforts aimed at emphasising functionally-driven ecosystem services.

## Introduction

The United Nations’ Decade on Biodiversity has recently ended (UN Resolution 65/161) and has shifted international focus towards restoration efforts (UN Resolution 73/284), yet a wide-spread biodiversity crisis continues to plague much of the planet^1–3^ with significant detrimental effects on marine ecosystems^4–6^. A vast ecological literature reaffirms that diversity in the marine realm (in the form of species richness, genetic variability, observed functional traits, etc.) is of great economic, scientific, and ecological value to mankind^7–9^. Biodiversity—simply considered here at the level of species richness and community composition—has also been deemed an ecosystem service in its own right for more intrinsic, abstract principles related to cultural, aesthetic, recreational, and existence value^8,10,11^.

Biodiversity losses are predicted with increasing homogenisation of marine soft-sediment systems due to elevated disturbance regimes^12^. Such losses are exacerbated by declines in available biogenic habitat. Increasing anthropogenic disturbance has led to the global decline of shellfish populations^13^, known to form large, complex beds that provide refuge and resources for other organisms^14^ and increase associated biodiversity due to increased habitat complexity^15^. Especially in soft-sediment habitats, complex biogenic reef structures provide hard surfaces and a three-dimensional structure above the sediment surface, increasing habitat diversity in areas otherwise comprised of sand and mud. This bed structure becomes extremely important in predominantly soft-sediment ecosystems like New Zealand’s Hauraki Gulf, where restoration projects utilising the green-lipped mussel (*Perna canaliculus*) are currently underway in response to their functional extinction due to anthropogenically-driven population collapse in the 1960s^16^.

Although negative impacts on community structure have been reported in instances where blue mussels outcompete other organisms for space and resources^17^, a vast majority of studies demonstrate that shellfish habitats increase species richness and diversity of macrofauna, epifauna, or fish as compared to nearby bare sediments^18–21^. In addition, it is known that a variety of coastal habitats (e.g. seagrasses, mangroves, kelp forests) serve as nursery grounds for commercially exploited species^22^. As a result of documented enhancement in fish recruitment and production, oyster reefs have been labelled ‘threatened nursey habitat’ in the United States^23^. While a potential nursey function of mussel habitats has been recognised^22^, the role of mussel restoration in augmenting this nursery function has yet to be fully resolved.

Fundamental ecological interest lies in determining how community structure might change based on species mobility. It is likely that mobile species (e.g. elasmobranchs and fishes) will differ from less mobile species (e.g. sea cucumbers, whelks, or infaunal worms) in their utilisation of restored beds. For post-larval life stages, mobility is often correlated with body size. Piscivorous fishes and elasmobranchs (observed up to 2.5 m in these coastal waters) are considered to be the most mobile species in these soft-sediment systems, likely to utilise mussel beds temporarily to consume reef residents^24^. While also capable of covering notable distances in short periods of time, some smaller reef fishes (blennies, triplefins) are known to establish home ranges and exhibit site fidelity upon location of preferential habitat^25^ and likely use mussel beds for greater periods of their lifetime. Mussel bed utilisation can also be tied to specific portions of the fish lifecycle (e.g., the documented association of juvenile snapper, *Chrysophrys auratus*, with *Atrina zelandica* beds/other structured estuarine habitats versus dramatic offshore movements during adulthood^26^). Together, these different lifestyle strategies make it difficult to predict changes in mobile fish community structure that may result from restoration efforts.

In contrast, smaller scales of mobility are observed in communities of epibenthic invertebrates (e.g. gastropod snails and sea cucumbers; 2–20 cm) which might take weeks to months to travel across estuaries in search of food or refuge, while comparable distances are achieved by mobile fish species in a fraction of this time. Similarly constrained by mobility, small encrusting invertebrate species (e.g. barnacles and ascidians; 1–10 cm) will settle on acceptable substrate throughout their adult life. The combined organic matter provisions and additional hard substrate provided by mussels likely attract members of this less-mobile, epifaunal community, although the strength of this effect across environmental gradients has yet to be resolved.

Macrofaunal communities composed mainly of polychaetes and molluscs typically exhibit minimal mobility and patchy distributions. Distinct macrofaunal assemblages have been identified beneath shellfish beds, dominated by species that thrive in high organic matter and hypoxic sediment conditions typified by mussel reefs^27,28^. Our restoration sites exhibit a range of sediment grain sizes, porosity, organic matter, and chlorophyll content, leading to context dependent impacts of mussel beds on sediment-dwelling communities.

Utilising sampling techniques with various levels of resolution, this study aims to document entire biological communities, containing elements separated by ranging capacity (mobile fishes and elasmobranchs, epifaunal invertebrates, and macrofauna) to determine how mussel restoration projects affect biodiversity. Surveys were repeated on multiple mussel beds and at nearby control soft-sediment locations without mussels, allowing for meaningful comparisons to be made between restoration locations while ultimately evaluating the ability of current mussel restoration projects to re-establish complex biological assemblages. We hypothesised that: 1. as mussel beds modify local environmental conditions experienced by resident organisms, shifts in community structure should be most evident at the least-mobile, macrofaunal scale (as opposed to changes seen in mobile fish communities); 2. mussel restoration would significantly enhance epifaunal communities as a result of additional settlement substrate above soft-sediments; and 3. variations to the sediment environment and spatial aggregation patterns observed in mussel beds would result in significant changes in community structure across restoration sites.

## Materials and methods

### Study area

**Figure 1:**
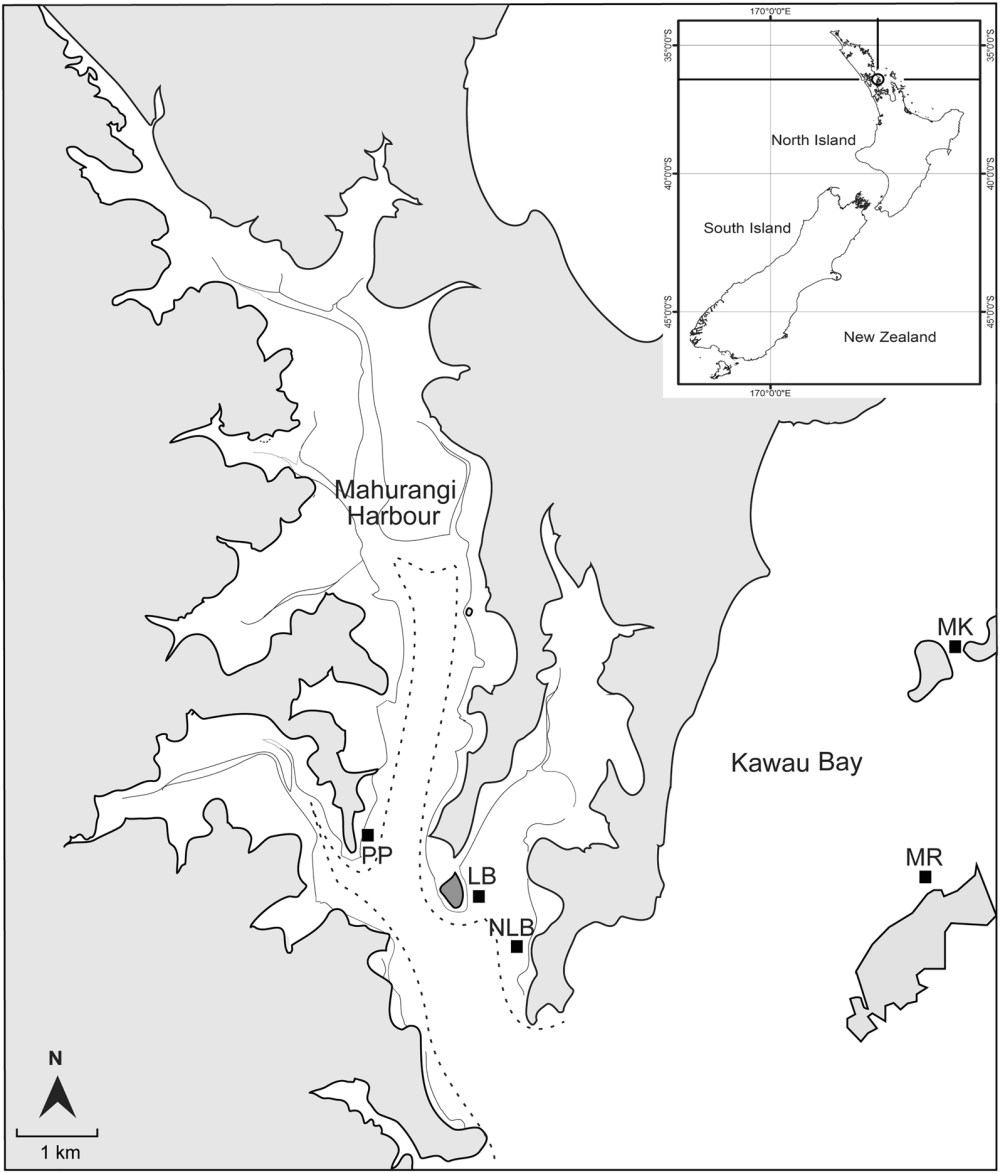
Each rectangle represents a paired study site, including a mussel bed and soft-sediment control (~ 5 m away), located either in Mahurangi Harbour or Kawau Bay, New Zealand. Pukapuka = PP, Lagoon Bay = LB, New Lagoon Bay = NLB, Motuora = MR, and Motoketekete = MK.

Between 2016 and 2019, adult green-lipped mussels (*Perna canaliculus*) were collected from mussel farms and transplanted to soft-sediment locations of similar depth (5–15 m). Chosen sites varied in terms of sediment composition, ranging from muddy sands to fine and medium sands (Table 1). Mussels were unloaded off a barge and sunk to the seafloor, creating multiple beds in the Hauraki Gulf on the north-eastern side of New Zealand’s North Island (Fig. 1). The majority of beds were created using 10–20 tonnes of mussels (~ 80–100 mm shell length) which clumped over time to form restored mussel beds ~ 20 to 30 m^2^ in size. Three beds were located near the mouth of Mahurangi Harbour (Pukapuka, Lagoon Bay, and New Lagoon Bay; PP, LB, and NLB respectively), and two were located adjacent to coastal islands in Kawau Bay (Motuora and Motoketekete; MR and MK). Beds displayed various spatial aggregation patterns at the time of study^29^, ranging from small clumps at Kawau Bay sites (~ 10 to 15 individuals m^2^) to generally larger clumps with smaller gaps at LB and PP (~ 25 to 75 m^2^). Restoration sites utilised in this study therefore varied in terms of observed sediment characteristics and mussel spatial aggregation patterns.

**Table 1:** Mean and range of environmental characteristics measured at each site, separated by restoration status (inside vs. outside mussel beds).

Site	Date established	Temp (°C)	Salinity (ppt)		Mud content (%)	Coarse sand (%)	SOM (%)	Chl *a* content (ug g^−1^)
PP	November 2018	21	33.3	In	21.9 (17.4–34.2)	1.3 (1.0–2.4)	3.5 (2.8–4.8)	8.3 (5.7–10.0)
				Out	21.4 (18.0–23.8)	1.1 (0.3–1.3)	2.9 (2.0–3.9)	5.7 (5.2–6.4)
LB	November 2018	21	32.6	In	22.8 (16.6–33.9)	0.7 (0.0–3.4)	3.9 (3.3–4.6)	6.6 (4.6–7.7)
				Out	22.2 (15.0–27.1)	0.5 (0.0–1.1)	2.9 (2.5–3.2)	4.5 (3.9–5.2)
NLB	July 2019	21	32.4	In	24.2 (14.6–44.1)	1.7 (0.7–4.4)	5.9 (4.1–8.0)	10.9 (5.2–14.0)
				Out	17.4 (13.7–20.6)	2.6 (0.3–5.0)	2.8 (2.5–3.2)	2.7 (1.5–3.3)
MR	November 2017	21	31.5	In	4.6 (2.3–7.1)	3.7 (1.9–4.4)	2.8 (2.5–3.3)	4.4 (2.9–6.4)
				Out	3.6 (2.7–6.3)	5.0 (2.4–7.5)	2.2 (1.8–2.6)	4.6 (3.4–6.1)
MK	October 2016	21	32.8	In	3.6 (3.2–3.7)	7.5 (6.4–8.7)	2.0 (1.8–2.1)	8.6 (8.2–8.9)
				Out	3.3 (2.7–3.9)	5.7 (5.1–6.2)	1.8 (1.7–1.9)	7.4 (7.0–7.9)

Mud is comprised of silt + clay (< 63 μm). Coarse sand > 500 μm. SOM = sediment organic material. Sites arranged from inner Mahurangi Harbour to outer Kawau Bay. MK sediment data from^30^. Pukapuka = PP, Lagoon Bay = LB, New Lagoon Bay = NLB, Motuora = MR, and Motoketekete = MK.

To investigate changes in diversity and biological community structure associated with restoration efforts, three separate methods were utilised at each site which sampled at scales relevant to the ranging capacities of each community of interest. Un-baited remote underwater video methods used to observe mobile species (fishes, sharks, rays) were conducted at all five beds. For logistical reasons, video transects and macrofauna cores (to visualise epifauna and macrofauna respectively) were conducted at four of the mussel beds (PP, LB, NLB, and MR). Methodologies were repeated ~ 5 m away from each mussel bed in nearby soft-sediments devoid of biogenic structure so that each site had a mussel bed and control location for comparison purposes. All surveys were conducted between November 2019 and February 2020.

### Un-baited remote underwater video methodology

To observe mobile species, 1–2 video recording devices (GoPro Hero7 Silver, resolution 1440p) were situated in the middle of each mussel bed and placed in an underwater housing system (height = 0.35 m). Such visual census methods are non-extractive, reduce size/species selectivity of other fish sampling techniques^31,32^, and avoid behavioural changes associated with diver surveys^33^. Recent fish community research on various Australian coastal habitats detected no assemblage differences between baited and un-baited video surveys^34^, and no bait was used in the present study. Our video cameras were connected to voltaic USB battery packs and set to record continuously during daylight hours. Resulting footage was trimmed so that all videos were the same length (just over 5 h) and each video started 1–2 h ahead of the turn of the tide.

To account for differences in water clarity between sites, a peg 14 cm high was inserted 0.75 m away from the face of the camera housing. Species that crossed in front of this peg were identified and their abundances recorded by Citizen Science volunteers. These volunteers were given the same instructions and training on underwater species identification. 3–5 independent volunteers coded each video. All data resulting from the same video were examined simultaneously for discrepancies in species identification, number of individuals observed, and position relative to the counting peg. Any discrepancies above 40% (e.g., more than 1/3 or 2/5 Citizen Scientists) were flagged and the time-point in question reviewed by the lead author to obtain a master dataset used in later analysis. Total abundance (the sum of all observed individuals of one species) and species richness (total number of taxa identified over the sampling period) were recorded and used in data analysis.

### Video transect methodology

To quantify benthic invertebrates and other epifaunal species, perpendicular transects, intersecting the bed centrally (c 20 m long), were placed across each restored mussel bed. With the aid of a plumb line (height = 20 cm) defining the distance from the camera lens to the reef, scuba divers videoed each transect with the camera lens at a 45-degree angle. For consistency, visible organisms from video footage were counted by a single, trained citizen scientist (the resulting dataset reviewed in its entirety by the lead author) and total abundances recorded for each transect.

Taxa were classified to family or species level where possible. Although frequently observed along video transects, species of small fish from the family Tripterygiidae (triplefins) were more easily seen in static videos and less likely biased by diver presence^33^ and were excluded in this portion of the analysis.

### Macrofaunal sampling methodology

At each mussel bed, eight sediment cores (10 cm diameter, 10 cm deep) were randomly taken along the longest transect used in the diver video sampling to obtain a representative sample of the macrofaunal community. Cores were sieved (500 μm mesh) and sieve contents preserved in 70% isopropyl alcohol and stained with rose bengal. Macrofauna were sorted and classified at the lowest taxonomic group possible.

To characterise sediment characteristics at each site, two small sediment cores (1.9 cm diameter, 3 cm deep) were collected next to each of the eight macrofauna cores. One sediment core was used for chlorophyll *a* and grain size analysis while the other was used for sediment porosity and organic matter content. All sediment samples were kept on ice in the dark and frozen at the laboratory until later analysis.

Percentage sediment organic matter (SOM) was determined by loss on ignition^35^. Sediments were left in a 60 °C oven until fully dried, and then weighed before and after combustion at 500 °C for 5 h. Sediment porosity was calculated as the difference between wet and dry weight (g), divided by core volume (cm^3^). Samples for sediment grain size (~ 20 g each) were digested with 6% H_2_O_2_ and rinsed after 48 h. Roughly 15 mL of 5% Calgon was added to each sample prior to analysis with a Malvern Mastersizer (ATA Scientific). Chlorophyll *a* was extracted from 1 g of freeze-dried sediment with 3 mL 90% acetone. Optical density of extracts were measured at 664, 665, and 750 nm with a UV–Vis spectrophotometer (Thermo Scientific, Multiskan Sky) before and after hydrochloric acid acidification (0.1 mL of 0.1 M HCl). Values at 750 nm were subtracted from values at 664 and 665 nm to correct for turbidity. Sediment chlorophyll *a* content was calculated using equations based on 90% acetone extraction^36^.

### Multivariate statistical analysis

Our strategy of sampling different size/mobility levels of seafloor biodiversity resulted in three separate multivariate data sets: mobile species data (from 1 to 2 remote videos at each mussel bed and control location), epifaunal species data (two diver transects for each mussel bed/control pair at sites PP, LB, NLB, and MR), and soft-sediment macrofauna data (from eight cores for each mussel bed and each control at sites PP, LB, NLB, and MR). Multivariate data consisted of counts from a total of 18, 18, and 88 taxa for un-baited remote underwater videos, transects, and macrofauna cores, respectively.

For all three levels of the biological community, we used permutational multivariate analysis of variance (PERMANOVA)^37^ to determine if community structure significantly varied by “Site” (PP, LB, NLB, MR, and MK) and by “Status” (mussel bed or control). For each dataset we created a Bray–Curtis similarity matrix calculated from square-root transformed abundance data and tested statistical significance using 9999 permutations of residuals under a reduced model (chosen significance level of α = 0.05). When Status (mussel bed vs. control) was a significant main effect or interaction term, similarity percentage analysis (SIMPER, One-Way Design; Factor = Status) was used to determine the percent of similarity of samples and to identify which species most highly contributed to observed differences between groups. Community data resulting from each of the three methods was visualised in multivariate space using nonmetric multi-dimensional scaling (nMDS), based on the same Bray–Curtis resemblance matrix constructed from the corresponding square-root transformed abundance data.

To determine if the best subset of environmental factors capturing multivariate macrofaunal assemblage structure varied between mussel bed and non-mussel bed locations, distance-based linear models (DISTLMs)^38^ were ran separately on resemblance matrices constructed from separate mussel bed and control community data. Our models utilised environmental data (SOM, chlorophyll *a*, porosity, percentage mud content and percentage coarse sand) as explanatory variables, and the community resemblance matrix (obtained using the Bray–Curtis similarity measure on square-root transformed abundance data) from corresponding macrofauna cores as the dependant variable. Parsimonious models were informed by Akaike information criterion (AIC) and constructed using a backwards elimination procedure. Predictor variables were log-transformed as necessary to meet assumptions of normality, and correlation among explanatory variables was examined; due to high correlation (R^2^ > 0.85) between percentage grain size parameters, percent medium sand was excluded from models. All multivariate statistical analyses were conducted in PRIMER v7^39^ with the add-on package PERMANOVA + ^40^.

### Univariate statistical analysis

The effects of Site (MK, MR, NLB, LB, and PP) and Status (mussel bed or control) on univariate measures of species richness (total number of species) and abundance (total number of individuals) were investigated for all three biological communities using 2-way ANOVA. Q-Q plots, Shapiro–Wilk tests, and Levene’s tests were used to check assumptions of normality and homogeneity of variance, and values log-transformed where necessary. Significance levels were set to *α* < 0.05, and standard errors (SE) were used to assess the precision of mean values. All ANOVA analyses were conducted using the R statistical package (version 4.1.0)^41^.

As we believed that the greatest changes in assemblage structure would be apparent in the macrofaunal community, we complimented our multivariate analysis of macrofauna with ranked species abundance plots. For each mussel bed and control, all eight replicate cores were pooled and the relative abundance (percentage of total abundance observed) plotted against the increasing log-ranked species^39^. This was done to visualise potential dominance of individual species, to compare relative evenness of macrofaunal communities, and to observe changes in the number of rare species (by comparing curve lengths) between sites. Dominance curves were created in in PRIMER v7^39^.

## Results

### Mobile species

**Figure 2:**
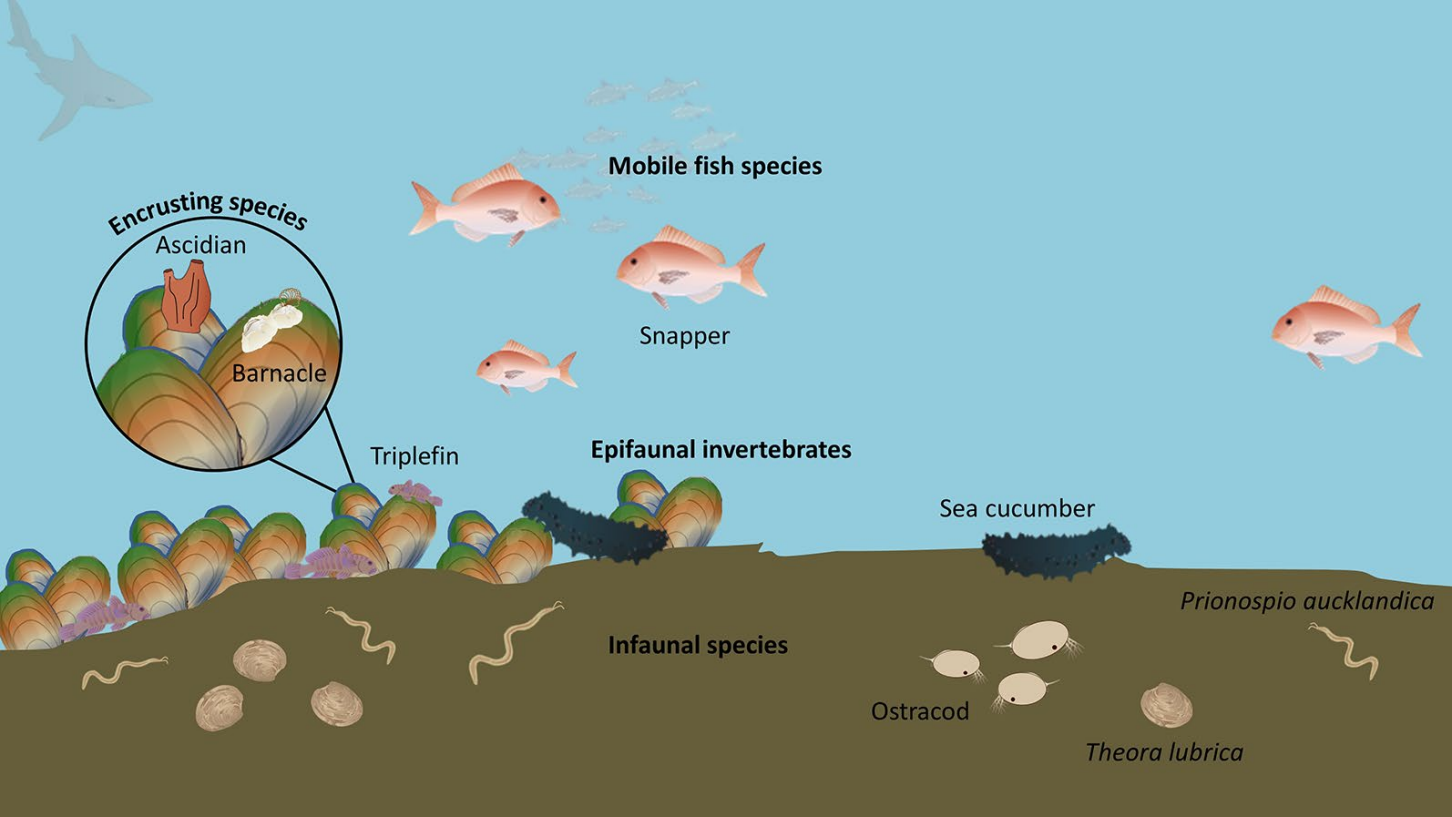
Conceptual diagram illustrating effects of subtidal mussel restoration on biological communities, differentiated by scales of mobility. Mobile communities of triplefins and commercially important fish species such as snapper (*Chrysophrys auratus*) are found in higher abundances on mussel beds than soft-sediment control sites, while highly transient elasmobranchs were not found to significantly differ with habitat type. Species richness and abundance of epifaunal invertebrates and encrusting species significantly increase with additional organic matter and hard substrate provided by mussels. Location-dependent changes in macrofaunal assemblage structure result from restoration efforts, with bivalve and polychaete detritovores more abundant beneath muddy, organically enriched mussel beds. Some symbols adapted from Integration and Application Network (ian.umces.edu/media-library).

**Figure 3:**
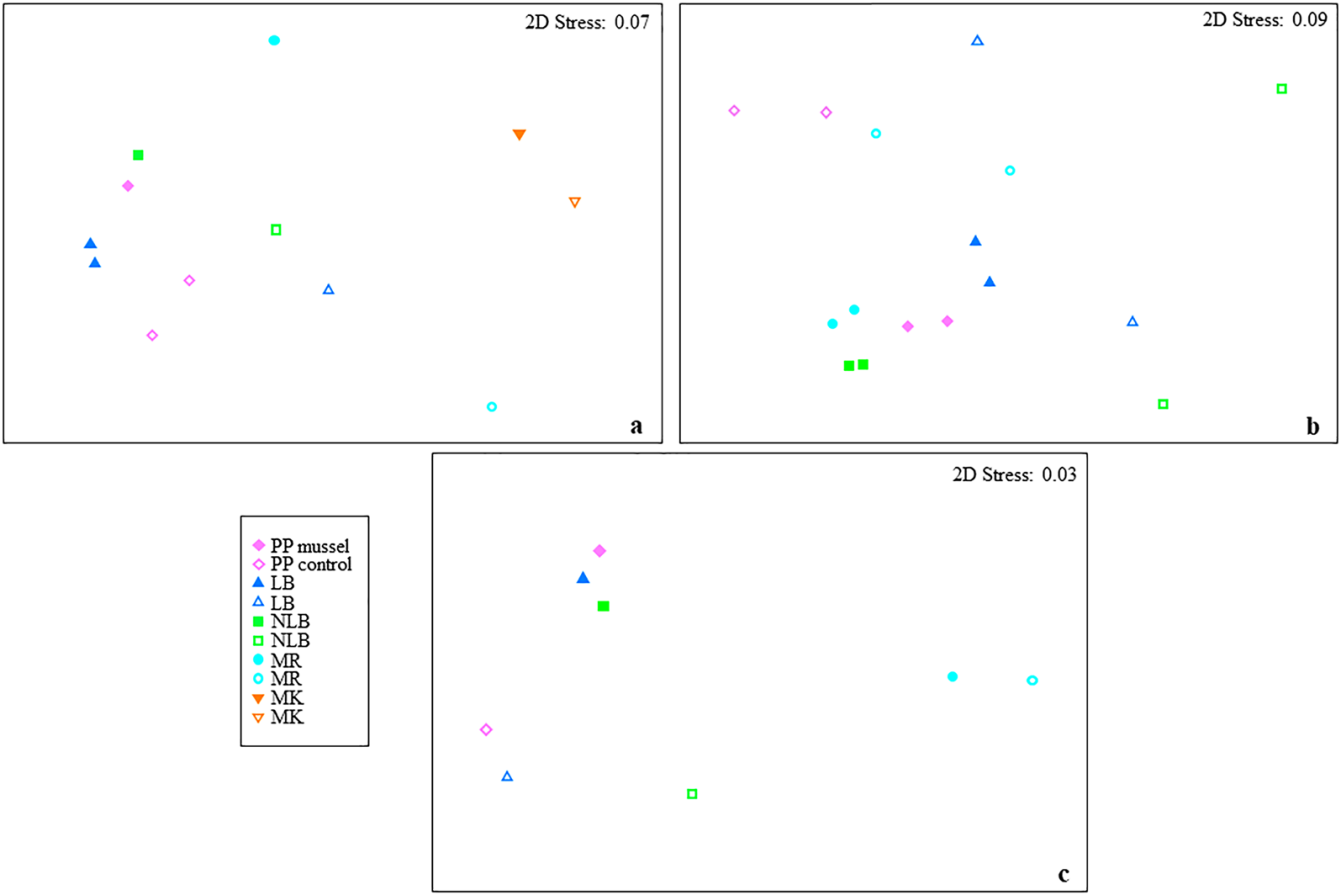
Two-dimensional non-metric multidimensional scaling (nMDS) plots for visualisation of differences in assemblage structure observed between sites (colour) and mussel bed status (fill). (**a**) Mobile community (points represent all available reps from un-baited video cameras); (**b**) epifauna/benthic invertebrate community (ordination showing two transects per site); and (**c**) macrofaunal community (calculated from the distance between centroids where n = 8 sediment cores for the combined factor SiteStatus). Ordinations created from Bray–Curtis similarity matrices on square-root transformed data using PRIMER v. 7 (Clarke & Gorley 2015; available at http://www.primer-e.com/).

Communities of mobile species utilising mussel beds were distinctly different from those found on nearby bare sediments (PERMANOVA; PsF_1,11_ = 4.40; *p* = 0.031; Table 2; Fig. 2). The nMDS ordination revealed a clear separation of mussel bed and non-mussel bed communities across a single axis (Fig. 3A). The interaction between Site and Status was also significant (PERMANOVA; PsF_4,11_ = 3.48; *p* = 0.027), indicating mussel bed effects were site dependent. SIMPER analysis revealed a substantial dissimilarity (62.94%) between mussel bed and non-mussel bed assemblages, a change largely driven by increases in snapper and triplefin abundance associated with mussel restoration (Supplementary Table S1). All six taxa identified as having a major contribution to this dissimilarity (triplefins, snapper, parore, mackerel, mullet, and trevally; Tripterygiidae, *Chrysophrys auratus*, *Girella tricuspidata*, *Trachurus* spp., Mugilidae, and *Pseudocaranx dentex* respectively) were found in higher abundances on mussel beds than in nearby soft-sediments.

**Table 2:** Permutational multivariate analysis of variance results based on the Bray–Curtis similarity measure for square-root abundance data of: (a) mobile species; (b) epifauna and benthic invertebrates; and (c) macrofaunal species.

Source	*df*	SS	MS	F	*P*
**(a) Mobile species**					
Site	4	12,872.0	3217.9	12.28	**0.0102**
Status	1	3974.0	3974.0	4.40	**0.0309**
Site × status	4	3652.7	913.2	3.48	**0.0265**
Residual	2	524.2	262.1		
Total	11	21,923.0			
**(b) Epifauna/benthic invertebrates**					
Site	3	14,343	4781.0	2.67	**0.0015**
Status	1	12,839	12,839.0	4.03	**0.0287**
Site × status	3	9548.9	3183.0	1.78	**0.0465**
Residual	8	14,338	1792.3		
Total	15	51,069			
**(c) Macrofauna**					
Site	3	48,559.0	16,186.0	11.03	**0.0001**
Status	1	7561.3	7561.3	3.27	0.0712
Site × status	3	6946.5	2315.5	1.58	**0.0079**
Residual	56	82,158.0	1467.1		
Total	63	145,220.0			

Statistically significant *p* values (α = 0.05) are bold. Tests conducted using 9999 permutations under a reduced model.

*MR* Motuora, *NLB* New Lagoon Bay, *LB* Lagoon Bay, *PP* Pukapuka.

The total abundance of mobile individuals observed on mussel beds was significantly (up to 20x) higher than off the beds (2-way ANOVA; F_1,2_ = 22.38; *p* = 0.042; Supplementary Table S3), with greatest abundances recorded within Mahurangi Harbour. An increase in species richness (the number of species observed) was also apparent with mussel restoration (2-way ANOVA; F_1,2_ = 62.74; *p* = 0.016), a pattern observed across all five sites (Table 3; Fig. 2).

**Table 3:** Summary of univariate diversity indices for all three community data sets.

Site	Status	Mobile species (un-baited remote videos)		Epifauna/benthic invertebrates (video transects)		Macrofauna (sediment cores)	
		Species richness*	Total abundance*	Species richness*	Total abundance*	Species richness	Total abundance
LB	Mussels	7.0	212.5	5.5 ± 0.5	46.5 ± 5.5	8.88 ± 1.29	28.13 ± 5.98
	Control	5.0	34.0	1.5 ± 0.5	3.0 ± 2.0	8.88 ± 0.91	18.13 ± 3.44
PP	Mussels	6.0	316.0	10.0 ± 0.0	229.0 ± 97.0	12.37 ± 0.96	35.13 ± 3.71
	Control	5.5	126.0	2.5 ± 1.5	7.5 ± 0.5	11.88 ± 1.17	27.00 ± 4.68
NLB	Mussels	9.0	353.0	5.5 ± 0.5	537.0 ± 32.0	10.00 ± 1.34	37.00 ± 5.30
	Control	6.0	49.0	1.0 ± 1.0	3.0 ± 3.0	15.25 ± 0.96	49.38 ± 10.82
MR	Mussels	9.0	149.0	11.5 ± 0.5	446.0 ± 19.0	25.50 ± 2.46	100.38 ± 14.18
	Control	3.0	7.0	5.0 ± 0.0	13.0 ± 3.0	26.13 ± 1.80	99.63 ± 11.05
MK	Mussels	6.0	16.0	n/a	n/a	n/a	n/a
	Control	5.0	6.0	n/a	n/a	n/a	n/a

Sites arranged over a decreasing mud gradient. Lagoon Bay = LB, Pukapuka = PP, New Lagoon Bay = NLB, Motuora = MR, and Motoketekete = MK. Asterisks (*) denote significant differences (2-way ANOVA, *p* < 0.05) between mussel beds and controls for the identified diversity index. Where applicable, data represent the mean ± SE.

### Epifauna and benthic invertebrate diversity

Encrusting invertebrates and large epifaunal species were rare in soft-sediments without mussels. A significant increase in total abundance—over 100-fold in some cases—was observed at mussel bed locations (2-way ANOVA; F_1,8_ = 32.24; *p* ≤ 0.001; Fig. 2; Table 3; Supplementary Table S4). Mussel restoration resulted in significant increases in species richness (albeit less substantial; up to fivefold) as well (2-way ANOVA; F_1,8_ = 119.12; *p* < 0.001). A total of 17 taxa were found across different mussel beds, while only 11 were found on control sediments.

Site and Status (bed vs. control) were both significant main effects in the PERMANOVA analysis (PsF_3,15_ = 2.67; *p* = 0.002; and PsF_1,15_ = 4.03; *p* = 0.029 respectively), suggesting that epifaunal communities on mussel beds were significantly different from those inhabiting surrounding bare sediments. A significant interaction between these main factors was also apparent (PERMANOVA; PsF_3,15_ = 1.78; *p* = 0.047; Table 2). These relationships are displayed in an nMDS plot (Fig. 3B), with transects from mussel bed communities markedly separated from controls. Communities from soft-sediment control locations were more spread apart in the ordination space, suggesting that the closely grouped restoration sites were, overall, more similar in assemblage structure. SIMPER analysis confirms that the similarity between mussel bed sites (47.23%) was much higher than the similarity between control sites (13.28%), and that species utilising the two habitat types were very different (88.76% dissimilar; Supplementary Tables S1 and S2). This difference was largely driven by encrusting species such as barnacles and ascidians, but all taxa which contributed to the observed dissimilarity between habitat types were found in higher abundances on mussel beds.

### Macrofaunal diversity

We found between 8.8 ± 1.3 and 25.5 ± 2.5 total macrofauna species at mussel bed locations, and 8.8 ± 0.9 and 26.1 ± 1.8 species at non-mussel bed locations. Species richness significantly changed with site (2-way ANOVA; F_3,56_ = 53.52; *p* ≤ 0.001), with more species found at MR than NLB, PP, and LB. The existence of mussel habitat, however, did not affect species richness (2-way ANOVA; F_1,56_ = 1.72; *p* = 0.195) or total abundance of counted individuals (2-way ANOVA; *F*_1,56_ = 0.71, *p* = 0.403) at the macrofaunal level.

PERMANOVA detected a significant interaction between Site and Status for macrofaunal assemblages (PsF_3,63_ = 1.58; *p* = 0.008). As reflected in the nMDS ordination, the way mussel beds affected macrofaunal assemblage structure varied by site; communities underneath beds at PP, LB, and NLB were distinctly different from their control counterparts, while the mussel bed assemblage at site MR—although highly distanced from PP, LB, and NLB in the ordination space—appeared to be more similar to its control sediments (Fig. 3C). A large number of species contributed to the overall dissimilarity (67.68%) between mussel bed and control sediments (Supplementary Table S1), with the bivalve mollusc *Theora lubrica* and polychaete worms from the family Spionidae notably responsible. Both taxa are well-accustomed to muddy, organically enriched sediments and were present in higher densities at mussel bed locations.

Differences in mud content (% < 63 μm) were apparent between sites (2-way ANOVA; *F*_3,56_ = 148.05, *p* < 0.001), with PP, LB, and NLB characterised by significantly muddier sediments than MR (Supplementary Table S5). Percentage coarse sand (> 500 μm) was significantly higher at MR than at PP and NLB (all of which were significantly higher than LB). SOM significantly increased from 2.70 ± 0.08% at control sediments to 4.01 ± 0.25% at mussel beds, although the strength of this effect varied with site (2-way ANOVA; *F*_3,56_ = 11.82, *p* < 0.001). Similarly, chlorophyll *a* content significantly varied with mussel bed status (2-way ANOVA; *F*_1,56_ = 55.45, *p* < 0.001), but effect direction and strength varied with site (2-way ANOVA; *F*_3,56_ = 22.11, *p* < 0.001).

DISTLMs captured 39.18% of the variation in macrofaunal community structure from soft-sediment control sites and 29.11% of the variation from mussel bed communities (Table 4). Sediment grain size characteristics (percent mud and coarse sand content) were important explanatory variables in models for both mussel bed and non-mussel bed communities, but chlorophyll *a* was selected in soft-sediment control models while SOM was retained in mussel bed models. The 10.07% decrease highlights that our chosen environmental parameters do a better job of explaining community variation in soft-sediment systems devoid of mussels.

**Table 4:** Summary table of distance-based linear model (DISTLM) results, showing chosen environmental predictors best fit to corresponding response communities.

Response community	AIC	Total variation explained (%) by chosen model	Predictors chosen	Pseudo-F	*P*
Mussel beds	243.03	29.11	Percentage mud content	7.20	**< 0.001**
			Log (coarse sand content)	4.32	**< 0.001**
			SOM %	4.17	**0.001**
Control sediments	237.82	39.18	Percentage mud content	12.15	**< 0.001**
			Log (coarse sand content)	6.68	**< 0.001**
			Chlorophyll *a*	1.96	0.059

Results shown are for models with lowest AIC values. Analyses based on Bray–Curtis similarity measures for square-root transformed macrofauna abundance data, constructed separately from mussel bed and control sediment cores.

*SOM* sediment organic matter.

Significant effects (*p* < 0.05) are shown in bold.

**Figure 4:**
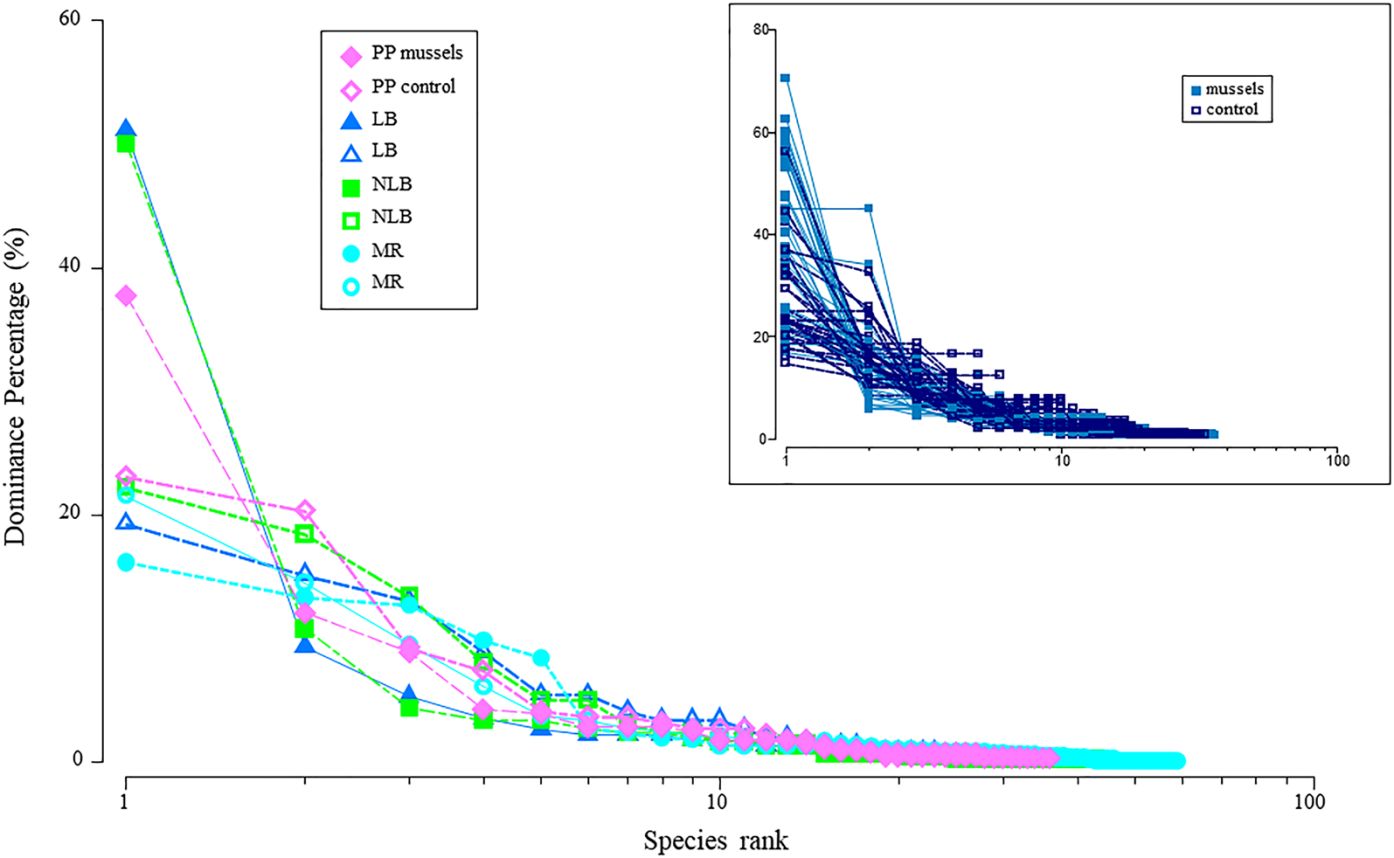
Ranked species abundance plot for pooled macrofauna cores (n = 8), split by site (colour) and mussel bed status (fill). Inset: dominance curves for all macrofauna cores (n = 64), split by mussel beds (light blue, filled) and soft-sediment controls (dark blue, open), highlighting increased dominance percentage in mussel bed communities. Labels for y-axis (relative abundance as a percentage of total abundance observed) and x-axis (species rank on log scale) are the same for both plots.

Ranked species abundance plots revealed differences in macrofaunal assemblage evenness across sites and by mussel bed status (Fig. 4). Control sites at PP, LB, and NLB displayed higher evenness (or lower dominance, as indicated by a lower, more gradual slope) than their mussel bed counterparts. MR was the only mussel bed exhibiting higher evenness than nearby control sediments. All other mussel bed communities (PP, LB, and NLB) appeared to be numerically dominated by one or two species which represented between 38 and 55% of the total sample population, while the percentage dominance of the most abundant species at bed MR was comparable to all controls (around 20%). Abundance plot lengths suggest that restoration had a minimal, variable effect on the number of rare species observed, which instead varied as a function of site (MR exhibiting the greatest number of rare species).

## Discussion

A majority of studies examining effects of benthic bivalves on associated species have focused on one aspect of the biological community^27,28,42,43^. This study documents changes in entire communities, highlighting that ranging capacity and site-specific environmental attributes differentially impact community structure at restoration sites. We documented significant changes in species richness, abundance, and assemblage structure in both mobile and epifaunal communities associated with subtidal, restored mussel beds, concluding that our restoration efforts have affected population and community level processes (summarised in Fig. 2). This in-situ study is first in reporting changes in species composition and assemblage structure on New Zealand’s subtidal restored mussel beds, while describing methodologies to be utilised in future monitoring efforts aimed at evaluating restoration progress against ecological objectives.

Epifaunal communities with minimal ranging capacity were found to be directly linked to mussel resources, both physically (with mussels as a source of habitat) and trophically (with mussels as a source of organic carbon and nitrogen). Extensive increases in species richness and diversity in epifaunal communities are likely a result of shell surfaces providing surface complexity, settlement substrate, and a source of refuge to colonisers^44^. Indeed, SIMPER analysis revealed that barnacles and ascidians (sessile and encrusting species almost never observed outside mussel habitat) highly contributed to the dissimilarity between mussel bed and non-mussel bed locations. Even for non-attached species, however, diversity increases in subtidal mussel beds have previously been documented^42^ and are a likely result of organic matter additions associated with bivalve biodeposition^14,20,45^. Sea cucumbers and small gastropod snails observed in this study were found in higher abundances on restored beds and also contributed significantly to the high dissimilarity (> 88%) between mussel and control locations. The constrained mobility of these species together with pronounced differences in measured diversity indices suggests a clear coupling of highly diverse epifaunal communities to restoration efforts. The fundamental link between restored beds and biodiversity enhancement at the epifaunal scale ultimately influences resource provisioning to communities at other organisational scales (as observed epifaunal species support a variety of regional reef predators)^46^ and is crucial in advancing public support and eventual upscaling of restoration efforts^47^.

The results of the current study demonstrate that mobile species—highly transient and (as opposed to the observed epifaunal communities) physically untied to mussel habitat—are disproportionately attracted to restoration sites compared to alternative soft-sediment habitats. Enhanced abundance of mobile species at restoration locations suggests that mussel beds are exploited for additional food resources, with highly mobile species utilising beds (albeit temporarily) to ingest both mussels and reef residents found in higher densities at restoration sites. High accumulations of mobile fish have been shown to deplete invertebrate communities in restored reefs elsewhere^48^, and such trophic interactions plausibly result in community changes documented at other organisational scales in this study. For example, increased triplefin abundance (up to 16 × higher on beds) may be partially responsible for lower overall abundances of known prey species (e.g., amphipods, ostracods) at restoration sites (Supplementary Table S1). Further examination of predator–prey relationships on restored beds is required to determine the potential effects of specific fish species on local prey populations and to predict changes in ecosystem function that result from modifications in community structure.

While the current study design does not allow us to distinguish between mechanisms driving observed community changes (e.g., food availability vs. refuge) the most notable enhancements at the mobile scale were observed in smaller species highly coupled to mussel bed habitat. Enhanced abundances of smaller fishes (triplefins, juvenile snapper) suggest decreased mortality as a result of refuges provided by structurally complex reef habitat. These ideas are supported by others who have tied triplefin success to physical complexity in the surrounding environment^49^ and snapper settlement to structured estuarine habitat types such as other bivalve beds in the Hauraki Gulf^26,50^. Other regional studies have shown the abundance of small, cryptic reef fishes is strongly influenced by habitat structural complexity and predator effects^51^, factors which likely impact abundance differences observed between mussel bed and non-mussel bed locations here. This study however does not resolve long-term survivorship of these species, which likely varies with life-style strategies. For example since resident demersal fish such as triplefins exhibit strong site fidelity^52^, these fish are likely associated with mussel beds throughout their lifetime, and documented increases in abundance likely reflect true population increases resulting from restoration efforts. In contrast, juvenile snapper (representing over 90% of total observed snapper in this study) were positively correlated to mussel restoration sites, but their long-term fitness and survivorship will be influenced by complex ontogenetic movements^26^ and human predation. Our results indicate that the structural complexity generated by restored beds is important in maintaining biodiversity at the mobile scale^53^, but acknowledge the current study represents a snapshot of the community at a specific time point; given the transient nature of mobile communities, further spatial and temporal variation can be resolved with future monitoring efforts.

All six mobile taxa identified to highly contribute to the dissimilarity between mussel beds and control sediments were found in higher abundances on restored beds, and four of these taxa (snapper, mackerel, mullet, and trevally; *Chrysophrys auratus*, *Trachurus* spp., Mugilidae, and *Pseudocaranx dentex* respectively) have well-established commercial value in New Zealand. Although increases in economically valuable fish species were evident on mussel beds, the current study design does not allow us to definitively differentiate between new fish production (those that exist solely because new habitat was generated) and those fishes that, as a result of behavioural preferences, merely aggregated around new structure without increasing fish production or abundance^54^. While difficult to quantify potential enhancement of fish production, Peterson and colleagues^43^ creatively combined growth and survivorship information from other published works to estimate that 10 m^2^ of oyster reef returns an additional 2.6 kg of mobile species annually. More recent studies predict slightly larger (but variable) increases in production (~ 4 kg per 10 m^−2^ year^−1^) as a result of increased fish recruitment on reefs^23^. We predict similar or even increased trends in fish production as a consequence of current mussel restoration efforts, as these beds replace important nursery habitats^50^ which have been severely degraded over the past decade. However, quantifying the magnitude and extent of this service was outside the current study’s scope.

The habitat heterogeneity hypothesis has been extended to bivalve systems^55–57^ and suggests that complex habitats support greater abundances and more diverse communities of macrofauna. Perhaps less anticipated, this was not the case for our restored beds, as species richness and total macrofaunal abundance remained largely unchanged as a result of restoration. Many studies report increases in macrofaunal diversity and abundance associated with bivalves^14,18,19,28,58^, but some note that changes in sediment composition associated with beds (increased organic additions leading to sediment anoxia, production of sulphides, etc.) favour small, opportunistic species perhaps less valuable in terms of their functional role in soft-sediment systems. While we did observe significant changes in sediment conditions as a result of mussel restoration (e.g. SOM, porosity, chlorophyll *a*), such opportunistic species (namely oligochaetes, capitellids) did not appear to highly contribute to community differences observed in this study (< 3% each; Supplementary Table S1).

Our ranked species abundance plots also suggest important compositional changes occur with mussel restoration despite traditional measures of macrofaunal diversity (richness, abundance) failing to differentiate ecologically relevant community changes between habitat types^59^. The three mussel beds in Mahurangi Harbour were numerically dominated by a few species (Fig. 4), typically the bivalve mollusc *Theora lubrica*, amphipods from the family Phoxocephalidae, and spionid polychaetes from the genus *Prionospio*. Detritivores and deposit feeders, these species thrive in muddy, organically enriched sediments typified by dense mussel beds and are important to local community-dynamics and sediment biogeochemistry^60,61^. In this study SOM inside mussel beds was found to be higher than in nearby soft-sediment controls, a likely result of biodeposition which supported higher abundances of these species, and is relevant to the delivery of other ecosystem services provided by restored beds^29^. It is notable that ranked species abundance plots suggest a more even community structure at MR, a site composed of individual mussels and small clumps as opposed to dense beds observed within Mahurangi Harbour (PP, LB, NLB)^29^. Together, these observations suggest that mussel aggregation patterns have functional consequences on macrofaunal communities and the services they deliver, which vary with local sediment conditions experienced.

Similar conclusions can be drawn from PERMANOVA results, which indicated the effect of mussel habitat on macrofaunal assemblage structure varied with restoration location. This interaction can be visualised in the corresponding nMDS plot (Fig. 3C), showing a large shift in community structure (observed along the vertical axis in 2D space) between mussel bed and non-mussel bed communities within Mahurangi Harbour (PP, LB, NLB) and a more modest shift in community structure (observed along the horizontal axis) for the mussel bed/control pair at the sandier MR site in Kawau Bay. This is surprising as one might predict that the creation/deposition of fine particles by mussels would result in greater infilling of interstitial spaces in coarse sands at MR, and thus have a greater impact on communities less acclimated to finer sediments (in contrast to macrofaunal communities already adapted to silty harbour sediments). These multivariate results should be viewed in tandem with community data at other organisational levels; unlike its macrofaunal assemblage, the epifaunal community established at MR is quite similar to inner-harbour sites (observed as a tight clustering of all mussel beds in multivariate space in the epifaunal data set; Fig. 3B). This suggests that a different mechanism drives community changes at the two organisational levels. At the epifaunal level, the addition of hard substrate results in colonisation by similar species, regardless of mussel bed location, while sediment modifications resulting from biodeposition affect macrofaunal communities in different ways depending on the local environmental conditions experienced. For example, it is possible that higher preservation of biodeposits occurs within the sheltered Mahurangi Harbour (where mussels have formed tightly packed, dense masses as compared to patchy Kawau Bay beds). These spatial differences likely influence organic matter available at the patch scale, which in turn affects macrofaunal assemblage structure. As community structure depends on the spatial configuration of biogenic habitat within the given environmental context, integration of spatial heterogeneity into future experimental designs will be pertinent in quantifying service value associated with varying macrofaunal assemblage structure.

The above insights are corroborated by the results of DISTLMs which suggest that SOM—shown here to be significantly higher within mussel beds—is a driving factor in altering macrofaunal communities. The 10% decrease in explanation of assemblage structure in mussel habitat is likely influenced by the site-dependent effect of mussel bed communities on macrofaunal assemblage structure. The way that increased SOM (as a consequence of mussel biodeposition) affects community structure is dependent on bed location and specific local environmental conditions (e.g. local hydrodynamics, sediment grain size, mussel aggregation patters, etc.), and creating a linear model which fully encompasses this interaction is expectedly more difficult. The DISTLM models include chlorophyll *a* concentration in the absence of mussels. As a proxy for the microphytobenthos, it seems intuitive that variations in the energetic foundation of most coastal food webs^62,63^ would be beneficial in the prediction of macrofaunal community structure. The substitution of chlorophyll *a* for SOM at mussel bed locations suggests the underlying macrofaunal community relies on biodeposition as a source of essential nutrients, and that other forms of organic matter will differentially affect community structure when biodeposits are unavailable. Others have separated the structural and functional role of mussels^58^ to determine that live mussels supply limiting resources (organic carbon and nitrogen) to sediment dwellers through biodeposition, which in turn increases the carrying capacity of these systems. Additional SOM inputs observed—while not shown to significantly increase macrofaunal diversity here—have been beneficial in the prediction of other ecosystem services associated with *P. canaliculus* restoration^29^.

Others have importantly noted the ‘dynamic nature of mussel bed structure’^64^. Bed structure can change as a result of mortality following extreme circumstances (e.g. severe weather events which can destroy entire beds), or more localised events (e.g. predation or dislodgement at the patch scale). In this study we were able to demonstrate significant changes in diversity across multiple scales of mobility at a specific time point; given the dynamic nature of beds through time, future changes in bed densities and the creation of additional restored beds will influence the magnitude and extent of observed community changes.

While mussel-associated communities can be influenced by local hydrodynamics^65^, it should be considered that, as ecosystem engineers, bivalves too can influence their environment (e.g. dampening wave energy, preventing sediment resuspension) even beyond the extent of reef boundaries^66^, which would have implications for community structure at larger spatial scales. We can conclude that such effects are likely limited to a scale of < 5 m in this specific case, as significant differences in community structure on and off beds were observed at this distance; however, it would be of interest to determine how engineering effects on infaunal community composition diminish at increasing distances from restored beds. In addition, others have shown that mussel size and/or bed age can influence species composition^67,68^; however, as these restored beds varied in age by less than 3 years and were composed of similarly sized individuals, such ideas were not explored further here.

Our findings substantiate the importance of complex structural features in enhancing overall diversity (species richness, abundance) in soft-sediment habitats. These subtidal mussels modified their physical environment in ways that differentially impacted associated biological community structure at various organisational scales. Community responses to restoration varied with species mobility and lifestyle strategies, and examining assemblage data separately allowed us to disentangle various mechanisms driving observed community changes. Most notable effects were derived from altered availability of resources; mussels generated organically enriched biodeposits which influenced local biogeochemistry and resultant macrofaunal communities directly tied to surrounding sediment conditions. Highly diverse epifaunal communities were supported by restoration, utilising mussels and their biodeposits for consumption and capitalising on hard substrate additions. Mussels and the epifaunal communities they supported then likely became a source of refuge and food to mobile species that supported predators at higher trophic levels. While we were able to link changes in community structure to the mobility of organisms at specified organisational scales, our results also highlight complex interactions between restoration effects and site selection on biological communities. Such context-dependency and strong location effects suggest that restored mussel beds should not be generalised as global hotspots of diversity^69^, and that critical site selection will influence biodiversity generated across scales of mobility. Determining the influence of subtidal mussel restoration on associated biological assemblages helps us better understand and evaluate ecosystem services underpinned by the diverse communities associated with restoration efforts.

## Supplementary Information

Supplementary Information.

## Data Availability

Datasets generated and analysed during the current study are available from the corresponding author on reasonable request.
