# Supplementary material for: Enhancing multiple scales of seafloor biodiversity with mussel restoration
Source: Sci Rep. 2022 Mar 23;12:5027. doi: 10.1038/s41598-022-09132-w (PMC8943123; doi:10.1038/s41598-022-09132-w)
Supplement: Supplementary file 1 — Supplementary Information. [file 41598_2022_9132_MOESM1_ESM.docx]

*The following supplement accompanies the article*

**Enhancing multiple scales of seafloor biodiversity with mussel restoration**

**Mallory A. Sea*, Jenny R. Hillman, and Simon F. Thrush**

*Corresponding author: msea579@aucklanduni.ac.nz

___________________________________________________________________________

**Table S1.** Summary of SIMPER results for mussel reefs and control sediments for all three methodologies, highlighting average abundance of discriminating species from each group, their percent contribution to the dissimilarity between groups, and the total cumulative percent contributions (cut off at 70%).

| **Mobile Species**  Un-baited Remote Underwater Videos  *Average Dissimilarity = 62.94 %* | | | | | | |
| --- | --- | --- | --- | --- | --- | --- |
|  | Abundance | | | Contribution % | | Cumulative % |
| **Species** | **Mussel Bed** | **Control** |  | |  | |
| Triplefins (Tripterygiidae) | 9.89 | 2.48 | 28.51 | | 28.51 | |
| Snapper (*Chrysophrys auratus*) | 5.29 | 4.67 | 13.84 | | 42.35 | |
| Parore (*Girella tricuspidata*) | 2.70 | 1.82 | 10.87 | | 53.22 | |
| Mackerel (*Trachurus* spp.) | 2.52 | 1.53 | 8.31 | | 61.53 | |
| Mullet (Mugilidae) | 1.82 | 0.00 | 6.26 | | 67.79 | |
| Trevally (*Pseudocaranx dentex*) | 1.25 | 0.47 | 6.21 | | 74.00 | |
| **Epifauna/ Benthic Invertebrates**  Video Transects  *Average Dissimilarity = 88.76 %* | | | | | | |
|  | Abundance | | | Contribution % | | Cumulative % |
| **Species** | **Mussel Bed** | **Control** |  | |  | |
| Barnacles | 13.37 | 0.00 | 39.53 | | 39.53 | |
| Seaweeds | 2.34 | 0.38 | 8.01 | | 47.53 | |
| Ascidians | 2.13 | 0.35 | 7.70 | | 55.23 | |
| Egg mass (likely gastropod) | 3.13 | 0.00 | 7.60 | | 62.83 | |
| Gastropods | 2.40 | 0.40 | 5.72 | | 68.54 | |
| Sea Cucumbers | 1.62 | 0.22 | 5.62 | | 74.17 | |
| **Macrofauna**  Sediment Cores  *Average Dissimilarity = 67.68 %* | | | | | | |
|  | Abundance | | | Contribution % | | Cumulative % |
| **Species** | **Mussel Bed** | **Control** |  | |  | |
| *Theora lubrica* | 2.88 | 1.61 | 7.69 | | 7.69 | |
| Ostracod | 1.87 | 2.44 | 6.06 | | 13.75 | |
| *Pseudopolydora* | 1.13 | 1.48 | 5.50 | | 19.24 | |
| *Prionospio aucklandica* | 1.21 | 0.46 | 4.03 | | 23.27 | |
| Phoxocephalidae | 1.35 | 1.46 | 4.00 | | 27.27 | |
| *Arthritica bifurca* | 0.45 | 1.01 | 3.54 | | 30.81 | |
| *Schistomeringos* | 0.56 | 0.79 | 3.17 | | 33.98 | |
| *Armandia maculata* | 0.97 | 0.50 | 3.14 | | 37.12 | |
| *Boccardia* | 0.29 | 0.76 | 2.91 | | 40.03 | |
| *Linucula hartvigiana* | 0.37 | 0.80 | 2.82 | | 42.85 | |
| *Cossura consimilis* | 0.72 | 0.71 | 2.61 | | 45.45 | |
| *Prionospio* spp. (other) | 0.62 | 0.40 | 2.55 | | 48.00 | |
| Oligochaeta | 0.53 | 0.57 | 2.45 | | 50.45 | |
| Lysianassidae | 0.31 | 0.66 | 2.29 | | 52.74 | |
| Nereidae | 0.45 | 0.52 | 2.26 | | 55.00 | |
| *Labiosthenolepis* | 0.33 | 0.54 | 2.24 | | 57.24 | |
| *Capitella* spp*.* | 0.48 | 0.45 | 2.18 | | 59.42 | |
| *Colurostylis lemurum* | 0.40 | 0.40 | 2.04 | | 61.46 | |
| Exogoninae | 0.35 | 0.60 | 1.96 | | 63.42 | |
| *Hemiplax hirtipes* | 0.16 | 0.42 | 1.83 | | 65.25 | |
| Lumbrineridae | 0.24 | 0.35 | 1.79 | | 67.04 | |
| Gastropod spp. (other) | 0.25 | 0.34 | 1.72 | | 68.76 | |
| *Aricidea* | 0.38 | 0.09 | 1.54 | | 70.29 | |

**Table S2.** Summary of SIMPER results for mussel reefs and control sediments for all three methodologies, highlighting average abundance of species that contribute to within-group similarity, their percent contribution to the similarity, and the total cumulative percent contributions (cut off at 70%).

| **Mobile Species**  Un-baited Remote Underwater Videos | | | |
| --- | --- | --- | --- |
|  | Abundance | Contribution % | Cumulative % |
| **Mussel Reefs**  *Average similarity = 44.29 %* |  |  |  |
| Triplefin (Tripterygiidae) | 9.89 | 48.03 | 48.03 |
| Snapper (*Chrysophrys auratus*) | 5.29 | 24.39 | 72.42 |
|  |  |  |  |
| **Soft-sediment Controls**  *Average similarity = 41.12 %* |  |  |  |
| Snapper (*Chrysophrys auratus*) | 4.67 | 49.08 | 49.08 |
| Triplefin (Tripterygiidae) | 2.48 | 31.36 | 80.43 |
|  |  |  |  |
| **Epifauna/ Benthic Invertebrates**  Video Transects |  |  |  |
|  | Abundance | Contribution % | Cumulative % |
| **Mussel Reefs**  *Average similarity = 47.23 %* |  |  |  |
| Barnacle | 13.37 | 53.51 | 53.51 |
| Seaweed | 2.34 | 12.77 | 66.28 |
| Sponge | 1.70 | 8.58 | 74.86 |
| **Soft-sediment Controls**  *Average similarity = 13.28 %* |  |  |  |
| Sponge | 0.50 | 33.36 | 33.36 |
| Fan worm spp. (other) | 0.83 | 32.12 | 65.48 |
| *Sabella spallanzanii* | 0.50 | 16.77 | 82.25 |
|  |  |  |  |
| **Macrofauna**  Sediment Cores |  |  |  |
|  | Abundance | Contribution % | Cumulative % |
| **Mussel Reefs**  *Average similarity = 34.89 %* |  |  |  |
| *Theora lubrica* | 2.88 | 33.04 | 33.04 |
| Phoxocephalidae | 1.35 | 12.71 | 45.74 |
| Ostracod | 1.87 | 12.34 | 58.09 |
| *Cossura consimilis* | 0.72 | 6.19 | 64.28 |
| *Prionospio aucklandica* | 1.21 | 5.71 | 69.99 |
| *Prionospio spp*. (other) | 0.62 | 3.06 | 73.05 |
|  |  |  |  |
| **Soft-sediment Controls**  *Average similarity = 35.11 %* |  |  |  |
| Ostracod | 2.44 | 19.26 | 19.26 |
| *Theora lubrica* | 1.61 | 15.37 | 34.63 |
| Phoxocephalidae | 1.46 | 9.09 | 43.73 |
| *Arthritica bifurca* | 1.01 | 8.22 | 51.95 |
| *Cossura consimilis* | 0.71 | 6.24 | 58.18 |
| *Linucula hartvigiana* | 0.80 | 5.21 | 63.40 |
| *Pseudopolydora* | 1.48 | 4.08 | 67.48 |
| Lysianassidae | 0.66 | 3.60 | 71.07 |

**Table S3.** Results of 2-way ANOVA showing the effects of Site and Status separately on measured diversity indices from un-baited remote underwater videos. Significant results (p ≤ 0.05) are indicated in **bold**. Analyses performed on untransformed data. Site labels: Pukapuka = PP, Lagoon Bay = LB, New Lagoon Bay = NLB, and Motuora = MR.

| **Factor** | df | SS | MS | F value | p-value |
| --- | --- | --- | --- | --- | --- |
| **Species Richness** |  |  |  |  |  |
| Site | 4 | 5.33 | 1.33 | 5.33 | 0.164 |
| Status | 1 | 15.69 | 15.69 | 62.75 | **0.016** |
| Site x status | 4 | 10.15 | 2.54 | 10.15 | 0.092 |
| Residuals | 2 | 0.50 | 0.25 |  |  |
|  |  |  |  |  |  |
| **Factor** | df | SS | MS | F value | p-value |
| **Total Abundance** |  |  |  |  |  |
| Site | 4 | 55776.00 | 13944.00 | 3.94 | 0.213 |
| Status | 1 | 79186.00 | 79186.00 | 22.38 | **0.042** |
| Site x status | 4 | 22462.00 | 5616.00 | 1.59 | 0.422 |
| Residuals | 2 | 7077.00 | 3538.00 |  |  |
|  |  |  |  |  |  |

**Table S4.** Results of 2-way ANOVA showing the effects of Site and Status (mussel reef or control) on measured diversity indices from video transects capturing epifauna and benthic invertebrate species. Significant results (p ≤ 0.05) are indicated in **bold**. Abundance data was log-transformed prior to analysis. Site labels: Pukapuka = PP, Lagoon Bay = LB, New Lagoon Bay = NLB, and Motuora = MR.

| **Factor** | df | SS | MS | F value | p-value |
| --- | --- | --- | --- | --- | --- |
| **Species Richness** |  |  |  |  |  |
| Site | 3 | 68.19 | 22.73 | 21.39 | **<0.001** |
| Status | 1 | 126.56 | 126.56 | 119.12 | **<0.001** |
| Site x status | 3 | 8.19 | 2.73 | 2.57 | 0.127 |
| Residuals | 8 | 8.50 | 1.06 |  |  |
|  |  |  |  |  |  |
| **Factor** | df | SS | MS | F value | p-value |
| **Total Abundance** |  |  |  |  |  |
| Site | 3 | 2.15 | 0.72 | 5.07 | **0.029** |
| Status | 1 | 4.56 | 4.56 | 32.24 | **<0.001** |
| Site x status | 3 | 0.22 | 0.07 | 0.51 | 0.687 |
| Residuals | 8 | 1.13 | 0.14 |  |  |
|  |  |  |  |  |  |

**Table S5.** Results of 2-way ANOVA showing the effects of Site and Status (mussel reef or control) on measured diversity indices and sediment characteristics from macrofaunal cores. Significant results (p ≤ 0.05) are indicated in **bold**. Abundance, SOM, Porosity, Mud, and Chlorophyll *a* data were log-transformed prior to analysis. Site labels: Pukapuka = PP, Lagoon Bay = LB, New Lagoon Bay = NLB, and Motuora = MR. SOM = sediment organic matter.

| **Factor** | df | SS | MS | F value | p-value |
| --- | --- | --- | --- | --- | --- |
| **Species Richness** |  |  |  |  |  |
| Site | 3 | 2692.00 | 897.30 | 53.52 | **<0.001** |
| Status | 1 | 28.90 | 28.90 | 1.72 | 0.195 |
| Site x status | 3 | 83.90 | 28.00 | 1.67 | 0.184 |
| Residuals | 56 | 938.90 | 16.80 |  |  |
|  |  |  |  |  |  |
| **Factor** | df | SS | MS | F value | p-value |
| **Total Abundance** |  |  |  |  |  |
| Site | 3 | 20.90 | 6.97 | 29.28 | **<0.001** |
| Status | 1 | 0.17 | 0.17 | 0.71 | 0.403 |
| Site x status | 3 | 0.95 | 0.32 | 1.33 | 0.274 |
| Residuals | 56 | 13.32 | 0.24 |  |  |
|  |  |  |  |  |  |
| **Factor** | df | SS | MS | F value | p-value |
| **SOM** |  |  |  |  |  |
| Site | 3 | 1.13 | 0.38 | 26.18 | **<0.001** |
| Status | 1 | 1.23 | 1.23 | 85.11 | **<0.001** |
| Site x status | 3 | 0.51 | 0.17 | 11.82 | **<0.001** |
| Residuals | 56 | 0.81 | 0.01 |  |  |
|  |  |  |  |  |  |
| **Factor** | df | SS | MS | F value | p-value |
| **Porosity** |  |  |  |  |  |
| Site | 3 | 0.90 | 0.30 | 9.91 | **<0.001** |
| Status | 1 | 0.07 | 0.068 | 2.25 | 0.140 |
| Site x status | 3 | 0.36 | 0.12 | 3.92 | **0.013** |
| Residuals | 56 | 1.69 | 0.03 |  |  |
|  |  |  |  |  |  |
| **Factor** | df | SS | MS | F value | p-value |
| **% Mud Content** |  |  |  |  |  |
| Site | 3 | 35.07 | 11.69 | 148.05 | **<0.001** |
| Status | 1 | 0.26 | 0.255 | 3.234 | 0.078 |
| Site x status | 3 | 0.23 | 0.077 | 0.978 | 0.410 |
| Residuals | 56 | 4.42 | 0.079 |  |  |
|  |  |  |  |  |  |
| **Factor** | df | SS | MS | F value | p-value |
| **% Coarse Sand** |  |  |  |  |  |
| Site | 3 | 129.92 | 43.31 | 26.50 | **<0.001** |
| Status | 1 | 3.19 | 3.19 | 1.95 | 0.168 |
| Site x status | 3 | 7.43 | 2.48 | 1.52 | 0.221 |
| Residuals | 56 | 91.51 | 1.63 |  |  |
|  |  |  |  |  |  |
| **Factor** | df | SS | MS | F value | p-value |
| **Chl *a*** |  |  |  |  |  |
| Site | 3 | 1.59 | 0.53 | 7.66 | **<0.001** |
| Status | 1 | 3.83 | 3.83 | 55.45 | **<0.001** |
| Site x status | 3 | 4.58 | 1.53 | 22.11 | **<0.001** |
| Residuals | 56 | 3.86 | 0.07 |  |  |
|  |  |  |  |  |  |
